# Strategies for simultaneous and successive delivery of RNA

**DOI:** 10.1007/s00109-020-01956-1

**Published:** 2020-11-04

**Authors:** Hanieh Moradian, Andreas Lendlein, Manfred Gossen

**Affiliations:** 1 Institute of Biomaterial Science, Helmholtz-Zentrum Geesthacht, Kantstr. 55, 14513 Teltow, Germany; 2 Berlin-Brandenburg Center for Regenerative Therapies (BCRT), 13353 Berlin, Germany; 3 Institute of Biochemistry and Biology, University of Potsdam, 14476 Potsdam, Germany

**Keywords:** Integrated co-transfection, Parallel co-transfection, Successive transfection, Co-expression, In vitro synthesized mRNA, Transfection methods

## Abstract

Advanced non-viral gene delivery experiments often require co-delivery of multiple nucleic acids. Therefore, the availability of reliable and robust co-transfection methods and defined selection criteria for their use in, e.g., expression of multimeric proteins or mixed RNA/DNA delivery is of utmost importance. Here, we investigated different co- and successive transfection approaches, with particular focus on in vitro transcribed messenger RNA (IVT-mRNA). Expression levels and patterns of two fluorescent protein reporters were determined, using different IVT-mRNA doses, carriers, and cell types. Quantitative parameters determining the efficiency of co-delivery were analyzed for IVT-mRNAs premixed before nanocarrier formation (integrated co-transfection) and when simultaneously transfecting cells with separately formed nanocarriers (parallel co-transfection), which resulted in a much higher level of expression heterogeneity for the two reporters. Successive delivery of mRNA revealed a lower transfection efficiency in the second transfection round. All these differences proved to be more pronounced for low mRNA doses. Concurrent delivery of siRNA with mRNA also indicated the highest co-transfection efficiency for integrated method. However, the maximum efficacy was shown for successive delivery, due to the kinetically different peak output for the two discretely operating entities. Our findings provide guidance for selection of the co-delivery method best suited to accommodate experimental requirements, highlighting in particular the nucleic acid dose-response dependence on co-delivery on the single-cell level.

**Electronic supplementary material:**

The online version of this article (10.1007/s00109-020-01956-1) contains supplementary material, which is available to authorized users.

## Introduction

Delivery of genes and other functional nucleic acids as a powerful tool for basic research [1–3], in biomedical/therapeutic applications and biotechnology [4–7], has been practiced intensely for decades. Corresponding methods have been constantly advanced [8–11], facilitating efficient cellular delivery of different types of nucleic acids such as plasmid DNA (pDNA), small interfering RNA (siRNA), small hairpin RNA (shRNA), and, more recently, single-guide RNA (sgRNA) as well as in vitro transcribed messenger RNA (IVT-mRNA) [9, 12–15]. For numerous applications, the simultaneous delivery of more than a single nucleic acid is advantageous or even mandatory. This includes co-delivery of multiple nucleic acids of the same type [16], multiple types of nucleic acids [14, 17], and a nucleic acid coordinated with another entity such as drug [18, 19] or protein [20]. Robust reliable co-delivery methods are, therefore, of critical importance in many gene transfer studies. Only few of these studies, however, focused on the applied co-delivery strategies and analyzed their impact on study’s outcomes, particularly for IVT-mRNA. Exemplary applications are the parallel transient overexpression of genes when required to analyze a given biological problem or even to realize the functional expression of the desired protein in the first place, e.g., as in the case of antibodies [21]. The coordinated knock-down (e.g., via siRNAs) and overexpression of related proteins are other instances [17, 22]. The co-transfection of traceable markers, mostly genes encoding fluorescent proteins, has been widely used to track, in particular on the single-cell level, the delivery of the gene of interest in a given study, as well as direct monitoring of gene transfer via in vivo imaging set up [23]. Placing two distinct functional entities described in these scenarios on separate vectors provides a level of experimental flexibility, which is difficult to achieve when combining them in a single vector. In some cases, however, the latter would not be possible, as delivery has to be performed at different time points, in order to coordinate peak expression of all transfected entities, which can kinetically vary from one nucleic acid type to the other [14].

**Fig. 1:**
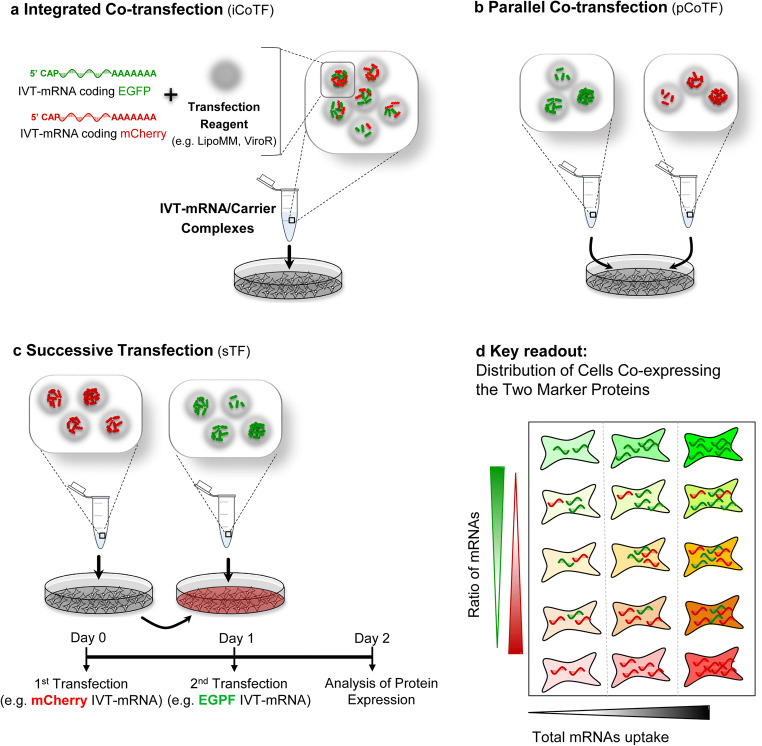
Schematic overview of different transfection methods: integrated co-transfection **a** refers to mixing different IVT-mRNAs prior to complexation with carrier, whereas in parallel co-transfections **b**, IVT-mRNAs are complexed in particles and added to cells separately, and in successive transfections **c**, cells are transfected with two types of IVT-mRNA with a 24-h interval. **d** Cellular uptake of different ratios of the two types of mRNA (vertical axis) and of different doses of both IVT-mRNAs (horizontal axis) results in different color distribution and intensity and can be used as a key readout for this study

Given this necessity for nucleic acid co-delivery in wide range of studies, and to fill this gap of crucial information, in this study, we investigated various strategies for co-delivery of nucleic acids, with a particular focus on IVT-mRNA. The aim of this study was to provide quantitative data to support proper choice of co-transfection methods and of experimental conditions within those methods. In this regard, we have investigated various co-delivery methods for simultaneous transfection, including “integrated co-transfection” (iCoTF) (Fig. 1a), “parallel co-transfection” (pCoTF) (Fig. 1b), as well as “successive transfection” (sTF) (Fig. 1c). The key readout was to determine heterogeneity and distribution of cells co-expressing both marker genes when using these methods in a dose-response manner (Fig. 1d). These studies were initiated in macrophages, which are a prime subject of our current research [24] and subsequently pursued in a cell line that is more readily available for routine transfection when compared with cells that had to be isolated and differentiated from primary human blood cells, at the cost of considerable time, effort, and resources. Co-transfection rates of IVT-mRNA in these cells were systematically investigated for different IVT-mRNA concentrations. In addition, various types of carriers, i.e., lipid- and polymeric-based carrier, were included to compare the different transfection methods. To further evaluate our concept, co-delivery and successive delivery of IVT-mRNA with another entity, i.e., siRNA with completely different properties and action mechanisms, was investigated. The findings of this study provide guidance to select the most effective method, depending on the specific experimental demands by identifying several critical criteria, of both, qualitative and quantitative nature.

## Materials and methods

### In vitro transcription of mRNA

Synthesis of mRNA coding enhanced green fluorescent protein (EGFP) and mCherry was performed via in vitro transcription, according to a previously published protocol [24]. Briefly, plasmid vectors, pRNA2-(A)_128_ [25], encoding EGFP, and pRNA2-(A)_128_-mCherry [24], both comprising a T7 promoter, 5′-UTR, the coding region for the respective fluorescent protein, head-to-tail duplicated human β-globin 3′-UTR, and followed by a 128-base polyadenine [poly(A)] sequence were linearized and purified by agarose gel electrophoreses using a gel extraction kit (Macherey-Nagel (MN), Germany). mRNAs were subsequently synthesized using a TranscriptAid T7 High Yield Transcription Kit (Thermo Fisher Scientific, Germany) following the manufacturer’s instruction. The 5’ end of IVT-mRNA was modified co-transcriptionally with anti-reverse cap analog (ARCA) (Jena Bioscience, Germany) [26]. Chemically modified IVT-mRNAs were generated by complete substitution of uridine and cytidine with 100 mM pseudouridine (Jena Bioscience, Germany) and 5-methylcytidine (m5C) (Jena Bioscience, Germany), respectively. IVT-mRNA was purified using lithium chloride precipitation and resuspended in UltraPure™ nuclease-free sterile water (Merck Millipore, Germany) containing 0.1 mM EDTA. The concentration of IVT-synthesized mRNAs was determined using a UV/Vis-spectrometer (NanoDrop 1000 Spectrophotometer; Peqlab, Germany) and further analyzed by denaturing agarose gel electrophoresis for integrity.

### Preparation of primary human monocyte-derived macrophages

Macrophages were derived from primary human monocytes as previously described [24]. Briefly, monocytes were purified from peripheral blood mononuclear cells (PBMC) isolated from buffy coats (Deutsche Rote Kreuz, Berlin; ethics vote EA2/018/16; Charité University Medicine Berlin) by negative selection using the Monocyte Isolation Kit II (Miltenyi Biotec, Germany) according to the manufacturer’s instruction. Purified cells were cultured in very low endotoxin (VLE) RPMI 1640 (Biochrom, Germany), supplemented with 10 vol% FBS (Sigma, Germany) and 50 ng mL^−1^ human macrophage colony stimulating factor (M-CSF) (Miltenyi Biotec, Germany) at 37 °C and 5 vol% CO_2_ for 6–7 days, with medium changes every third day. At the end of the differentiation period at day 7, the medium was replaced with warm VLE RPMI supplemented only with 10 vol% FBS.

### Co-delivery of mRNA to primary human macrophages

Macrophages were transfected using chemically modified IVT-mRNAs in combination with Lipofectamine MessengerMAX (LipoMM; Thermo Fisher Scientific, Germany). Co-delivery was performed as follows:

- Integrated co-transfection

MessengerMAX reagent was diluted in Opti-MEM medium (Gibco, Germany) at 1:50 volume ratio and incubated for 10 min at RT. The resulting solution was added to the equal volume of premixed EGFP and mCherry mRNAs diluted in Opti-MEM medium to a final mRNA concentration of 4 ng μL^−1^ (Fig. 1a).

b Parallel co-transfection

The diluted MessengerMAX reagent was divided in two equal parts, each was separately added to the equal volume of either EGFP or mCherry mRNA solutions (Fig. 1b). The LipoMM-mRNA mixtures were briefly vortexed and incubated for 5 min at RT for complex formation. Respective volumes of the co-transfection mixtures were transferred to each well to deliver the final mRNA concentrations equal to 12.5, 40, 125, and 250 ng mL^−1^ in cell culture medium. Cells were analyzed for fluorescent protein expression 24 h after transfection. Viability of cells was evaluated via 4′,6-diamidine-2′-phenylindole dihydrochloride (DAPI) staining, and quantified via flow cytometry, considering DAPI-negative cells as live cells. For the transfection conditions, chosen viability was above 95% throughout. Macrophages transfected with mRNA only or carrier only did not show any discernible fluorescent signal nor a reduction in viability, as previously shown by us [24].

In both conditions, cells were transfected in 6-well plate format, with a density of 2.00E + 06 cells per well in 2 mL complete RPMI medium.

### Co-delivery of mRNA to HeLa cells via a lipid- or a polymeric-based carrier

HeLa (ATCC; CCl-2) cells were seeded at a density of 3.00E + 05 cells per well in 6-well plates, in high glucose DMEM, supplemented with GlutaMAX™, pyruvate (Gibco, Germany), 10 vol% FBS (Biochrom, Germany), and 1 U mL^−1^ penicillin-streptomycin (Gibco, Germany), 24 h before transfection. LipoMM-mRNA co-transfection mixtures (both for iCoTF and pCoTF) were prepared exactly as described in the previous section; see “Co-delivery of mRNA to primary human macrophages” aside from using non-modified mRNAs.

A polymeric-based transfection reagent was also investigated by using Viromer RED (ViroR; Lipocalyx, Germany). To prepare ViroR-mRNA complexes for iCoTF, equal amounts of EGFP and mCherry non-modified mRNAs were diluted in 318 μL of the provided ViroR buffer at final concentration of 11 ng μL^−1^. In another tube, 1.25 μL of Viromer® reagent was placed on the tubes’ wall and immediately mixed with 30 μL of the dilution buffer and vortexed for 5 s. The buffer containing premixed IVT-mRNAs was then added to the diluted Viromer® solution, mixed swiftly, and incubated for 15 min at RT. In parallel, two individually prepared mRNA solutions of EGFP or mCherry in 160 μL dilution buffer were separately mixed with 15 μL of diluted ViroR for pCoTF.

Corresponding volumes of the co-transfection mixture were transferred to each well of 24-well plate to deliver final IVT-mRNA concentration of 660, 330, 110, and 33 ng mL^−1^ in 500 μL culture medium, for both lipid-based and polymeric-based transfection reagents. Cells were further evaluated by fluorescent microscopy and flow cytometry 24 h after transfection.

To evaluate HeLa cell viability, cells were seeded in 96-well plate at a density of 1.40E + 04 cells per well, 24 h prior to transfection. Subsequently, transfection was performed with either the highest, i.e., 660 ng mL^−1^ or the lowest, i.e., 33 ng mL^−1^, concentrations of mRNA in 100 μL medium per well. Mock transfection was done by addition of carrier only to the corresponding wells. The viability assay was performed 24 h upon cell transfection using Cell Titer® 96 AQ_ueous_ Non-Radioactive MTS Assay (Promega, Germany), according to manufacturer’s instruction. Briefly, 20 μL of MTS mixture (MTS solution mixed with PMS solution at ratio of 20:1) was added to each well and incubated for 3 h at 37 °C. Cells were treated with 100 μL of 1 mM CuCl_2_ as positive control, i.e., maximum cell death. The plates were protected from light at all steps. Absorbance was measured at wavelength of 490 nm using a SpectraMax M5 microplate reader (Molecular Devices, San Jose, CA).

### Co-delivery of plasmid DNA (pDNA) in HeLa cells

Plasmids were transfected using a transfection grade 25 kDa linear polyethylenimine (PEI) (Polyscience, Warrington, PA). To prepare the co-transfection mixture, PEI was dissolved in 150 mM NaCl solution to a final concentration of 2.4 mM. The diluted PEI solution was either added entirely to the equal volume of premixed pDNA solution in 150 mM NaCl (40 ng μL^−1^) for iCoTF or splitted and mixed separately with each of the two pDNAs, for pCoTF. The resulting mixture was vortexed for 30 s and incubated for 10 min at RT for complex formation. HeLa cells, pre-seeded 24 h before transfection at a density of 2.50E + 05 cells per well of 6-well plates in 2 mL culture medium, were transfected with 1320, 660, 220, and 66 ng μL^−1^ of pDNAs and analyzed for gene expression 48 h after transfection.

### Successive delivery of IVT-mRNA

HeLa cells were seeded at a density of 8.00E + 04 cells per well of 12-well plates in 1 mL culture medium, 24 h before first transfection. Non-modified IVT-mRNA coding mCherry was initially transfected via LipoMM in various doses (660, 330, 110, 33 ng μL^−1^), according to previously described protocol at day 0; see “Co-delivery of mRNA to primary human macrophages” section. Cells were transfected for the second time with identical doses of mRNA coding EGFP at day 1. Cells were evaluated for fluorescent protein expression at day 2.

### Co-delivery versus successive delivery of siRNA and IVT-mRNA

**Fig. 2:**
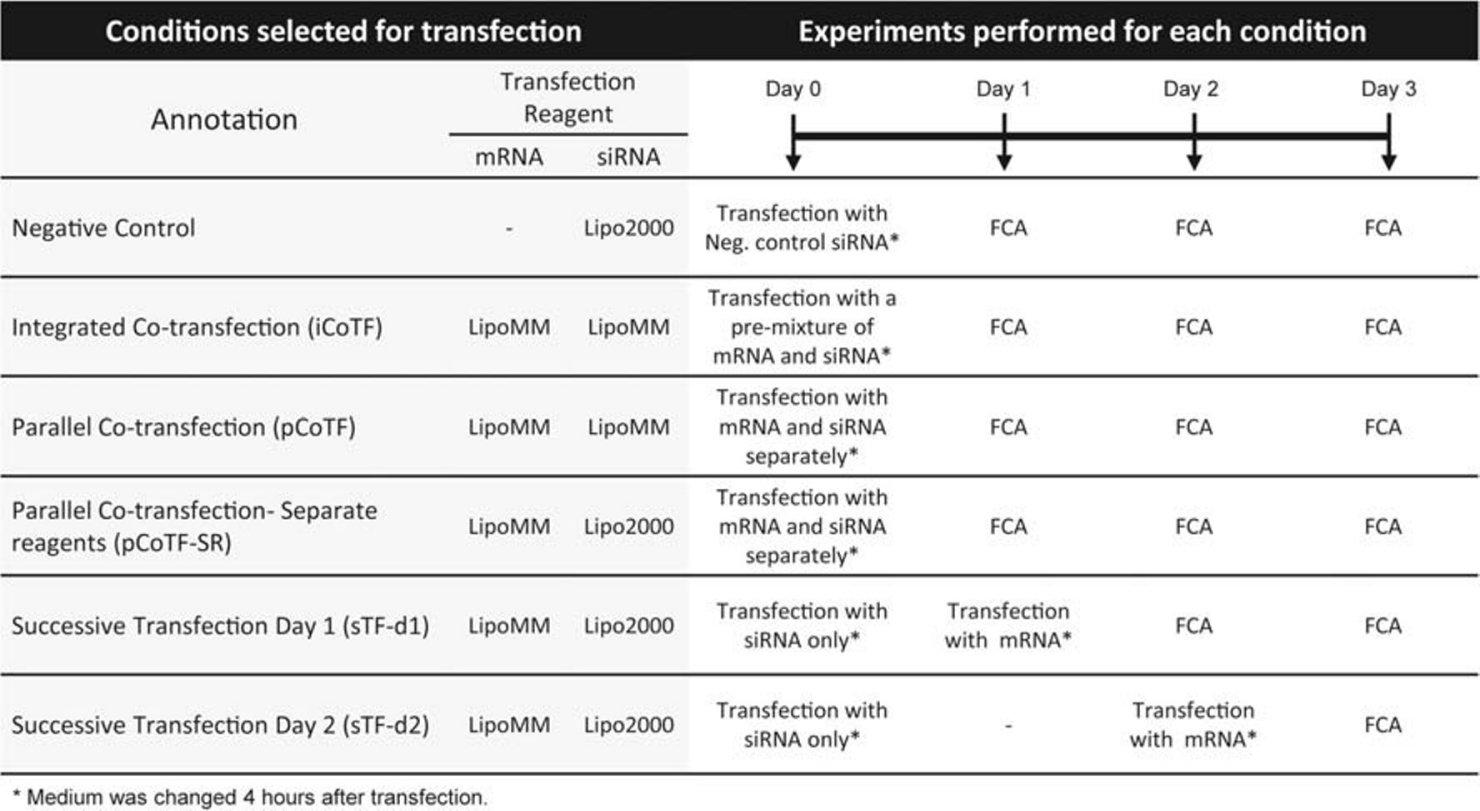
Design of experiments and description of transfection conditions for co-delivery of IVT-mRNA and siRNA; (FCA, flow cytometry analysis; LipoMM, Lipofectamine MessengerMax; Lipo2000, Lipofectamine 2000)

HeLa cells stably expressing a destabilized EGFP in a homogenous fashion [27] were seeded at a density of 3.00E + 04 cells per well of 24-well plates, in 500 μL complete DMEM medium (10 vol% FBS, no antibiotics), 24 h before transfection. Co-transfection conditions for simultaneous or successive delivery of siRNA and non-modified IVT-mRNA are outlined in Fig. 2.The preparation procedure is described as follows. To prepare iCoTF mixture, LipoMM reagent was diluted in Opti-MEM medium at 1:50 volume ratio and incubated for 10 min at RT. A premixed solution of IVT-mRNA coding mCherry (200 ng) and 20 pmol Stealth™ RNAi EGFP reporter control (Invitrogen, Germany) in Opti-MEM medium was added to the diluted LipoMM solution at 1:1 volume ratio, mixed well, and incubated for 5 min at RT for complex formation. Similarly prepared LipoMM solution was added separately to each of IVT-mRNA or Stealth™ RNAi solutions for pCoTF. Lipofectamine 2000 (Lipo2000; Thermo Fisher Scientific, Germany) was exclusively used for Stealth™ RNAi delivery in pCoTF-SR as well as all successive transfections, according to manufacturer’s protocol; see Fig. 2. Briefly, Lipo2000 was diluted at 1:50 volume ratio in Opti-MEM medium and incubated for 5 min at RT. Twenty picomole of Stealth™ RNAi EGFP reporter control or the negative control was diluted in equal volume of Opti-MEM medium, mixed with Lipo2000 solution, and incubated for 20 min at RT for complex formation. A single transfection mixture of LipoMM-IVT-mRNA coding mCherry was prepared as outlined above and added either simultaneous with Lipo2000-siRNA mixture at day 0 or upon siRNA transfection at days 1 and 2 for pCoTF-SR, sTF-d1, and sTF-d2, respectively. Medium was changed 4 h after transfection for all conditions.

### Evaluation of transfection efficiency by fluorescent microscopy

To evaluate the fluorescent proteins (EGFP and mCherry) expression, cells were imaged via a Nikon inverted microscope ELIPSE T*i*-U equipped with long-life mercury light source, Intensilight C-HGFI, with single-band filter sets, Semrock filter GFP-BP (466/40, 525/50 nm) for green fluorescence, or Semrock filter TRITC (562/40, 641/75 nm) for red fluorescence (mCherry) observations. The NIS-Elements imaging software package (version 4.51) and Image J software were utilized to analyze microscopic images.

### Quantitative analysis of transfection efficiency by flow cytometry

Cells were harvested at previously specified time points by TrypLE Select (Gibco, Germany) according to manufacturer’s instruction. Upon washing with cold flow cytometry washing solution (PBS pH 7.2, BSA, EDTA), cells were analyzed with a MACSQuant VYB® flow cytometer (Miltenyi Biotec, Germany). All flow cytometric data were analyzed with FlowJo software V10.

### Statistics

Data are presented as means ± standard deviation (SD) of at least three independent experiments. Multiple comparison *t* test was performed via a GraphPad Prism 7.00 (La Jolla, CA 92037, USA). Statistical significance (alpha) was defined as 0.05.

## Results

### Co-delivery of IVT-mRNA in primary human monocyte-derived macrophages

Two different methods were investigated for IVT-mRNA delivery in monocyte-derived primary human macrophages. For iCoTF, lipoplexes were prepared by premixing of EGFP and mCherry IVT-mRNAs before adding to the transfection reagent, whereas for pCoTF, the independently formed complexes were added to the same well (Fig. 1a, b). To evaluate the co-transfection efficiency for each method in dependence of the delivered mRNA doses, percentage of double-positive cells and intensity of reporter genes expression were recorded via fluorescent microscopy and quantified via flow cytometry.

**Fig. 3:**
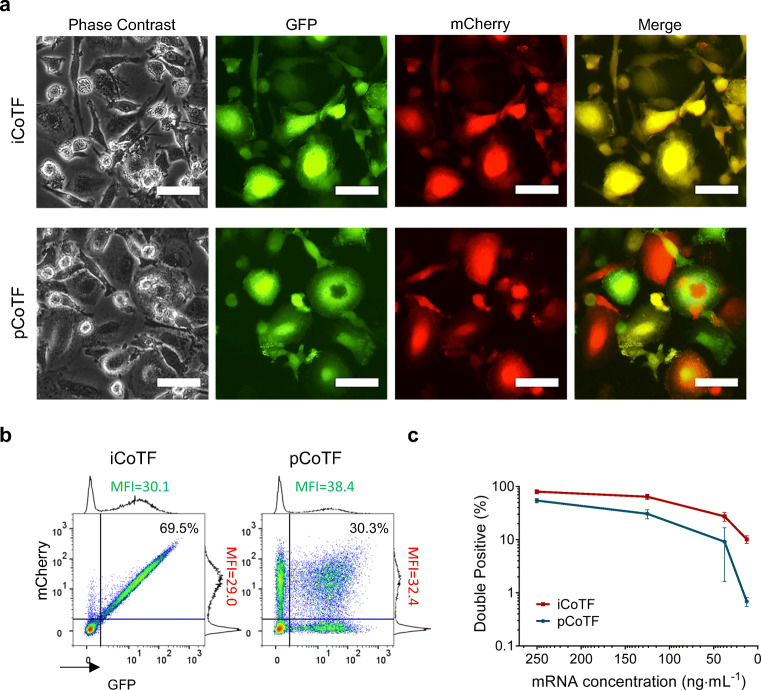
Simultaneous transfection of macrophages with EGFP and mCherry coding IVT-mRNA with two different strategies. Fluorescent microscopy images (bar = 50 μm) **a** and flow cytometric density plots **b** depicting the fraction of double-positive cells in integrated versus parallel co-transfected macrophages for an mRNA dose of 125 ng mL^−1^. Analogous measurements were performed for cells transfected with various mRNA doses **c**. All experiments were performed using a liposomal carrier (LipoMM); multiple comparison *t* test revealed significant difference between iCoTF and pCoTF for all evaluated mRNA doses (*p* < 0.05). No fluorescent signal-/double-positive event was observed for untransfected macrophages. Values are presented as mean ± SD, *n* ≥ 3. Error bars indicate SD. (iCoTF, integrated co-transfection; pCoTF, parallel co-transfection)

There was an obvious difference in transfection patterns in macrophages transfected with two co-delivery methods even for high mRNA dose (125 ng mL^−1^) as shown in Fig. 3a. The difference was also remarkable on single-cell level, presented as density plots in Fig. 3b, with iCoTF resulting in almost all of the transfected cells equally expressing both marker genes at the same level. In contrast, pCoTF results in heterogeneous population of cells expressing different levels of each reporter gene, observed as different color spectrum in merged fluorescent image (Fig. 3a) and the wide distribution of cells within double-positive gate (Fig. 3b). pCoTF also resulted in slightly higher level of fluorescent protein expression compared with iCoTF, measured via mean fluorescent intensity (MFI) of the peak on the adjacent histograms. Decreasing mRNA doses, however, drastically reduced the rates of the double-positive cells transfected with pCoTF method (ca. 50-fold) and compared with a moderate decrease (ca. 7-fold) with iCoTF (Fig. 3c).

### IVT-mRNA Co-delivery methods: cell type and carrier dependence

The results obtained for macrophages in the course of ongoing studies [24] were repeated in HeLa cells to analyze whether or not the initial observations for the co-transfection experiments were limited to a specific cell type and also were extended by testing different carrier types. To this end, different doses of IVT-mRNAs coding mCherry and EGFP were co-transfected in HeLa cells using an example of a liposomal (LipoMM) and a polymeric (ViroR) gene carrier in side-by-side experiments. The encapsulation efficiency of each carrier system was evaluated for different carrier to mRNA ratios, as explained in “Supplementary Methods.” The ratios carrier/mRNA used throughout the experiments shown revealed more than 95% encapsulation efficiency for LipoMM and more than 87% for ViroR, both of which confirms the successful entrapment of mRNA within carrier (Supplementary Fig. 1). Moreover, the physicochemical properties, i.e., size and zeta potential measurement of particles prepared with different types of mRNA (EGFP and mCherry), were measured (Supplementary Table 1). Comparison of different particles revealed no significant differences between carrier complexes prepared with different types of mRNA both for LipoMM and ViroR.

**Fig. 4:**
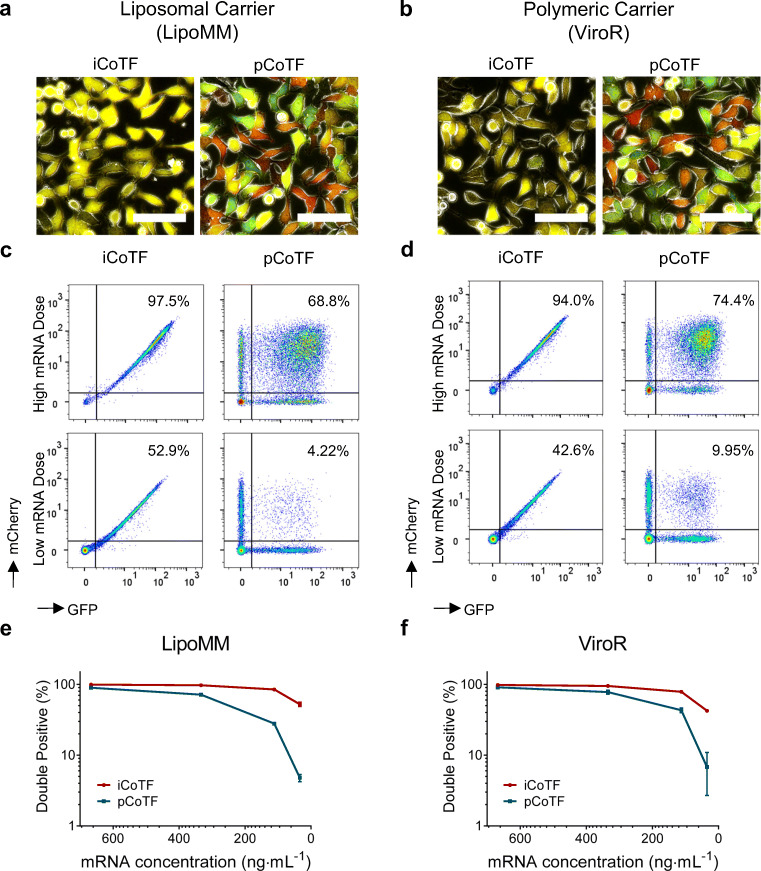
Comparison of IVT-mRNA co-delivery using liposomal and polymeric carriers in HeLa cells. Fluorescent microscopy images of HeLa cells co-transfected with non-modified IVT-mRNA coding mCherry and EGFP (330 ng mL^−1^) with LipoMM **a** and ViroR **b** (bar = 100 μm). Density plots of integrated versus parallel co-transfection for high mRNA doses (330 ng mL^−1^; upper panels) and low mRNA doses (33 ng mL^−1^; lower panels) for HeLa cells transfected with LipoMM **c** and ViroR **d**. Percent of double-positive cells plotted against descending mRNA concentrations compared within two various co-transfection approaches both for LipoMM **e** and ViroR **f**. Multiple comparison *t* test revealed significant difference between iCoTF and pCoTF for all evaluated mRNA doses for both LipoMM and ViroR (*p* < 0.05). No fluorescent signal-/double-positive event was observed for untransfected and mock-transfected HeLa cells, i.e., cells treated with carrier only, as well as mRNA only. Values are presented as mean ± SD, *n* ≥ 3. Error bars indicate SD. (iCoTF, integrated co-transfection; pCoTF, parallel co-transfection)

The fluorescent images showed that regardless of the type of carrier, iCoTF always resulted in higher percentage of cells expressing both EGFP and mCherry. Noteworthy, the overall expression levels were higher in LipoMM than for ViroR for both fluorescent proteins, as depicted in Fig. 4a, b.

Evaluation of the co-delivery rates at single-cell level by flow cytometry indicated the similar diagonal pattern for iCoTF and a rather wide distribution within double-positive cells for pCoTF mediated by both LipoMM and ViroR. The direct comparison of these two strategies revealed that the rates of double-positive cells are higher for integrated than for parallel co-transfection (Fig. 4c, d), particularly notable when lower doses of mRNA were transfected (Fig. 4e, f). There are no indications that these effects are either cell type or carrier dependent.

Cell viability measurement was performed using MTS assay. There was no significant difference between viability of cells transfected with iCoTF and pCoTF methods at the lowest and highest concentration of mRNA. Besides, mock transfection, i.e., cells transfected with transfection reagents only, and mRNA only had no significant impact on cell viability (Supplementary Fig. 2).

**Fig. 5:**
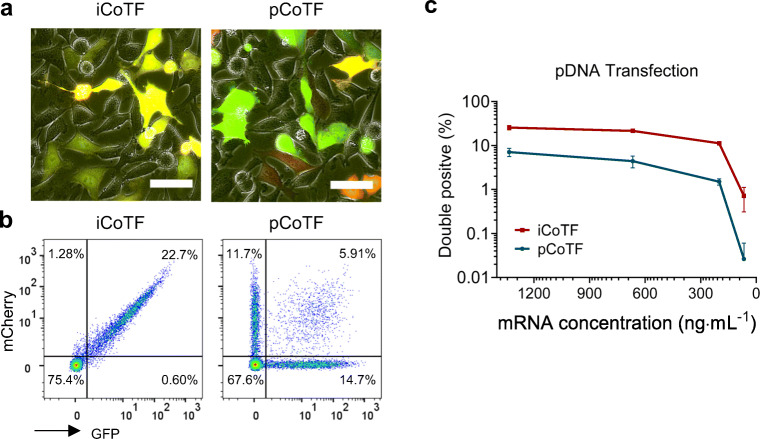
Comparison of integrated and parallel co-transfection approaches for pDNA delivery in HeLa cells using PEI; fluorescence microscopy images illustrating EGFP and mCherry channel merged with phase contrast (bar =50 μm) **a**, flow cytometric density plots **b**, and percent of double-positive cells plotted against descending pDNA concentrations **c**; multiple comparison *t* test revealed significant difference between iCoTF and pCoTF for all evaluated pDNA doses (*p* < 0.05). No fluorescent signal-/double-positive event was observed for untransfected HeLa cells. Values are presented as mean ± SD, *n* ≥ 3. Error bars indicate SD. (iCoTF, integrated co-transfection, pCoTF, parallel co-transfection)

For comparison, HeLa cells were also transfected with the template plasmids used in IVT. This is possible as both fluorescent protein encoding plasmids contain a strong CMV promoter, allowing DNA-directed transgene expression. Linear PEI was chosen as a well-established carrier for pDNA transfection. As shown in Fig. 5, comparative analysis of the two co-delivery strategies for pDNA resulted in transfection patterns similar to those observed for IVT-mRNA.

### Successive delivery of IVT-mRNA

**Fig. 6:**
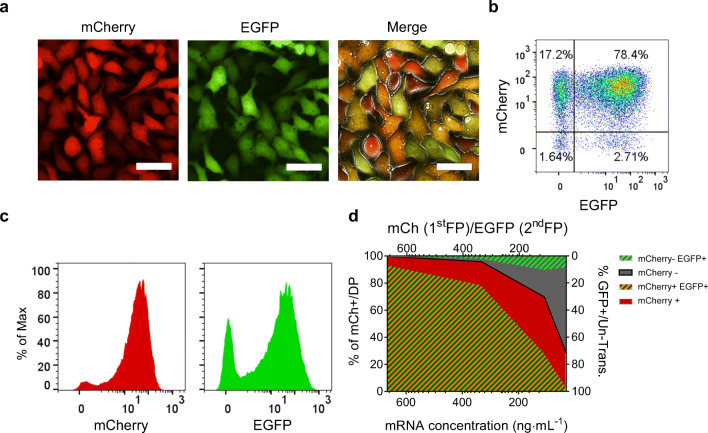
Successive transfection of IVT-mRNA in HeLa cells with LipoMM; fluorescent images depicted as single fluorescence channels (left, middle), as well as merged with phase contrast in the right panel (bar =50 μm) **a**, flow cytometric analysis of cells via density plots **b**, and histograms of mCherry and EGFP **c**; summary of different populations’ frequencies plotted for cells which were sequentially transfected with various mRNA doses **d**; no fluorescent signal was observed for untransfected and mock-transfected (carrier only) HeLa cells. Values are presented as mean, *n* ≥ 3

In order to create and investigate a model for successive transfection of IVT-mRNA, HeLa cells were transfected in separate steps as depicted in Fig. 1c. There was a clear difference in both transfection efficiency and fluorescent protein intensity between the first and the second transfection, which resulted in a remarkable heterogeneity in expression patterns of the two transgenes, particularly noticeable in merged fluorescent image (Fig. 6a). Interestingly, the overall ratio of double-positive to single-positive cells was very similar to what was observed earlier for parallel co-transfection; however, the ratio of the two single-positive cell populations was substantially different, when analyzed via flow cytometric density plots (compare Figs. 4c to 6b).

Despite the high transfection rates for mCherry (95 ± 1.2%), the first fluorescent protein, only 81.4 ± 4.1% of cells were positive for the EGFP reporter transfected second (Fig. 6b, c). Interestingly, those cells not transfected in the 1^st^ round appeared to be more resistant to the 2^nd^ round of transfection when compared with those already successfully transfected. Both effects in combination resulted in the example given in Fig. 6b in about 6-fold more mCherry+/EGFP- cells when compared with cells only positive for EGFP; for overall quantification, see also Fig. 6d. At decreasing doses of IVT-mRNA, this effect was even more pronounced due to the increased percentage of 1^st^ round-negative cells. A quantitative evaluation is also provided in Table 1. The same IVT-mRNAs (identical batches) were also transfected in control experiments in a reverse order, i.e., EGFP first and subsequently mCherry. This allowed us to rule out that the observed bias resulted from quality differences in the IVT-mRNA preparations. Results are shown in Supplementary Fig. 3, underpinning that the order of transfection is decisive.

**Table 1:** Quantitative evaluation of IVT-mRNA transfection

mRNA concentration (ng mL^−1^)	1st transfection	2nd transfection		
	% mCherry+ (of total cells)	% EGFP+ (of total cells)	% EGFP+ within mCherrry+	% EGFP+ within mCherry-
660	99.8 ± 0.1	93.6 ± 2.3	93.8 ± 2.2	Not reported
330	95.7 ± 1.2	81.4 ± 4.1	82.3 ± 3.9	61.0 ± 2.5
110	69.7 ± 0.5	39.9 ± 6.1	41.8 ± 6.2	35.4 ± 6.2
33	28.4 ± 1.1	13.4 ± 0.5	15.4 ± 0.7	12.5 ± 0.8

### Co-delivery versus successive delivery of siRNA and IVT-mRNA

The co-delivery of distinct types of nucleic acids, here siRNA and mRNA, was exemplary evaluated for the different transfection strategies described here, using fluorescent proteins as proxy readouts. A cell line stable expressing EGFP with nearly 100% expression penetrance [27] was transfected simultaneously with a siRNA knocking down the endogenous fluorescent EGFP and introducing a mCherry IVT-mRNA as a transfection marker.

Various strategies for simultaneous or successive delivery of siRNA and IVT-mRNA were studied and compared in side-by-side experiments. The goal was not only to achieve the highest percent of cells transfected with both siRNA and mRNA but also to achieve respective maximum efficiencies. This is of particular importance due to the kinetically different highest effectiveness of these two distinct entities, i.e., about 24 h for mRNA expression versus 60–72 h for siRNA knock-down. Therefore, three strategies, namely, “integrated co-transfection,” “parallel co-transfection,” and “parallel co-transfection-SR” were selected for simultaneous delivery. Both RNAs were transfected with LipoMM in the first two methods, whereas in the third approach, mRNA was transfected with LipoMM and siRNA with Lipo2000. In parallel, “successive transfection-d1”and “successive transfection-d2” were done, in which mRNA was transfected 1 day or 2 days after siRNA transfection, respectively (Fig. 2).

**Fig. 7:**
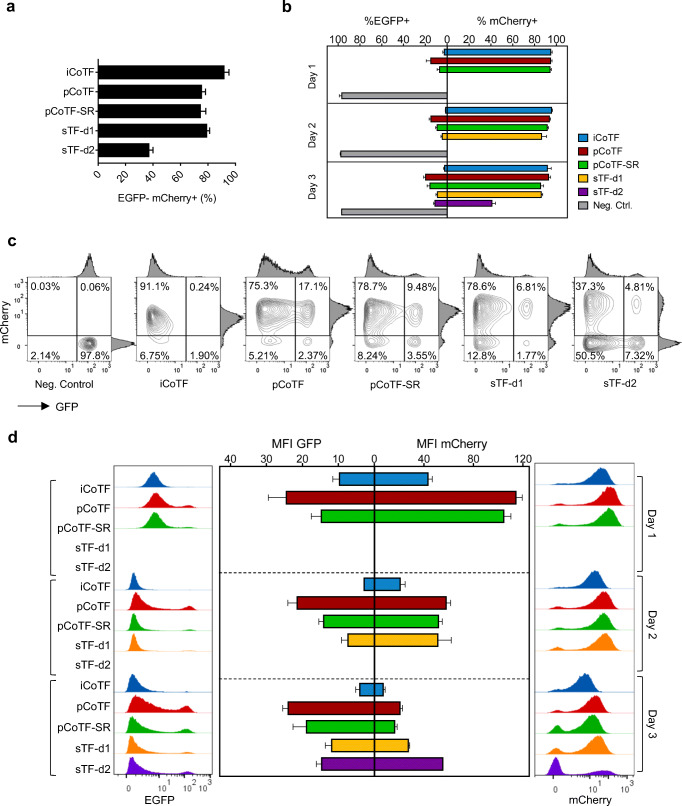
Co-delivery versus successive delivery of siRNA and IVT-mRNA in d2EGFP HeLa cells transfected with different conditions (see Fig. 2 for more detailed information) evaluated by flow cytometry; **a** percent of EGFP^−^mCherry^+^ cells plotted for comparison within different groups, **b** kinetic of mCherry expression (percent of mCherry^+^ cells) as well as EGFP knock-down (reduction in percent of EGFP+ cells) for different conditions over 3 days, **c** density plots along with adjacent histograms of cells at day 3, **d** kinetic of EGFP knock-down level depicted as EGFP mean fluorescent intensity (MFI) in parallel with mCherry expression level (MFI calculated for mCherry^+^ cells) indicated for different methods over 3 days (middle panel) shown side by side with EGFP (left panel) and mCherry (right panel) histograms; values are presented as mean ± SD, *n* ≥ 3. Error bars indicate SD

A substantial difference was observed in mCherry expression level at day 3, when different co-delivery methods were compared. Precisely, the maximum level of mCherry expression in transfected cells when analyzed microscopically was correlated to sTF-d1 and sTF-d2. There were few EGFP+ cells in parallel and successive transfected cells (Supplementary Fig. 4). iCoTF and sTF-d1 resulted in the highest percent of EGFP^−^mCherry^+^ cells’ population (Fig. 7a, c). When investigated over the course of 3 days, the same pattern was observed consistently for different groups compared in terms of mCherry^+^ versus EGFP^+^ within each day (Fig. 7b). However, the highest EGFP knock-down was detected for sTF-d1 and iCoTF with the smallest EGFP-positive population (Fig. 7c) as well as the lowest EGFP intensity (Fig. 7d).

The kinetics of EGFP knock-down was tracked over 3 days indicating a minimal EGFP intensity over days 2 and 3 (Fig. 7d). Nevertheless, a slight increase was noticeable at day 3, when compared with histograms of the same samples at day 2 (Fig. 7d, left panel). Within each day, the lowest EGFP expression was correlated to the iCoTF and the sTF-d1 among all samples (Fig. 7d).

The kinetic study of mCherry over 3 days was depicted either as mean fluorescent intensity (MFI) of mCherry plots or as histograms (Fig. 7d, right panel). At day 1, the maximum mCherry intensity was observed for pCoTF and pCoTF-SR but not iCoTF. The same pattern of the highest mCherry intensity for pCoTF was also observed at day 2 and interestingly for sTF-d1 and sTF-d2 at day 3 (Fig. 7d, middle panel). As expected, the correlation between two transgenes at single-cell level decreased drastically for sTF-d2 (Fig. 7). Overall, the results of this experiment for co-delivery of two distinct entities such as siRNA and IVT-mRNA demonstrate that there is no superior approach per se*,* but decisions on the transfection strategy have to be made according to the specific experimental demands. For instance, if co-transfection efficiency for both entities is the main interest, iCoTF is recommended. In contrast, when the reasonable rates of knock-down concurrent with the highest overexpression level (intensity) are primarily desired, here, sTF-d2 is the method of choice.

## Discussion

Comparison of integrated versus parallel co-transfection methods for simultaneous co-delivery of IVT-mRNA coding for spectrally distinct fluorescent proteins was investigated. Our findings show remarkable differences in rates of co-transfected cells and the level of reporter gene expression between the two methods. Specifically, integrated co-transfection of mRNA resulted in almost identical expression levels of the two proteins in a given transfected cell, whereas parallel co-transfection led to heterogeneous population of cells in terms of transgene expression even within double-positive cells population. This effect was strongly dose dependent; in other words, the differences between these approaches were more pronounced for low mRNA doses. Thus, integrated co-transfection could be the method of choice, especially when low amounts of nucleic acid are required, or as suggested by Xie et al., in case of restricted transfection efficiency [28]. Besides, they reported that only the integrated and parallel co-transfection methods significantly affected the results, but not the other process parameters such as cell density and ratio between two transgenes [28].

Explanations for the striking differences between iCoTF and pCoTF in their ability to mediate a high proportion of cells simultaneously taking up two (or more) distinct nucleic acids as observed by us and others [2, 28–30] can be readily explained by considering either the number of cargo-loaded nanoparticles taken up by a given cell or the number of nucleic acid molecules per carrier unit. For the latter, high numbers of nucleic acid molecules per nanoparticle are expected to attenuate the expression heterogeneity when simultaneously delivering, e.g., two distinct nucleic acids. Provided that these are loaded on the carrier in the same efficiency, a high number of nucleic acids delivered per particle virtually ensure that even with a low number of particles taken up by the cell, both nucleic acid species would be present. It should be noted here that the decisive numbers in quantifying any such effect are not the numbers of nucleic acids per particle as measured directly in analytical settings but rather the number of those nucleic acids functionally delivered in the cell. In this respect, mRNAs should be a preferred model of research, as compared with pDNA as they do not have to overcome the barrier of the nuclear membrane and would be functionally available directly in the cytoplasm, minimizing the number of unknowns in such calculations. A similar consideration applies for the number of carriers taken up per cell; high numbers are expected to attenuate heterogeneity, also in the case of pCoTF. In order to provide an exemplary idea about the absolute numbers involved, one can reasonably estimate the number of mRNA molecules per cell based on the average number of 350 IVT-mRNA molecules per lipoplex particle, given by Leonhardt and colleagues, and considering the number of particles which is taken up by each cell. Using advanced microscopic methods, a maximum of 15 lipoplex particles were observed in each transfected cell for a relatively high dose of mRNA (1 μg mL^−1^) [31]. These numbers have successfully been used to model especially kinetic aspects of mRNA delivery, from carrier uptake to protein expression [31–34].

The observed difference in expression heterogeneity between the iCoTF and pCoTF strategy was independent of the carrier system used, i.g. lipoplex or polyplex. However, given their diverging—and still controversial—intracellular trafficking routes and cargo release mechanisms [29, 35, 36], there might be ways to tailor carriers to accommodate task for co-delivery in a more directional manner, e.g., for polymeric carriers by controlling their size and cargo density [35–37].

The notable heterogeneity of co-transfected cells limits the utility of parallel co-transfection method in addressing scientific or technological questions, where the co-expression of two proteins (or regulatory RNA) in the same cell is mandatory. For other applications, though, such a heterogeneity is instrumental and can be exploited accordingly. One elegant example is the recent application of the simultaneous transfection of multiple plasmids, analogous to our parallel co-transfection, referred to as “poly-transfection method” by Gam and colleagues [30]. In their study, the resulting heterogeneity in the expression of transfected genes was analyzed on the single-cell level for the fast and efficient characterization and optimization of synthetic genetic systems and circuits [38–40]. Functional relevance of the integrated co-transfection method has been addressed in a study by Mendia et al., suggesting the highest cartilage matrix deposition secreted by integrated co-transfected human chondrocytes expressing both IGF-I and SOX9 [41].

For several applications, delivery of two nucleic acids to the same cells has to be successive over time. Examples are experimental settings, where expression of the first nucleic acid is prerequisite for the proper and/or effective function of the second nucleic acid. To mimic such a scenario, the successive delivery of mRNA coding mCherry followed by mRNA coding EGFP was evaluated. Results indicated that overall transfection efficiency was lower for the second than for the first transfection. A closer analysis of the fluorescent patterns revealed that the initially transfected cells had a higher probability for re-transfection when compared with those cells that were not transfected in the first round. One can speculate that this is the result of a functional heterogeneity in the cell population, rendering a subtraction of cells more resistant to nucleic acid uptake. This finding is of particular interest for studies where frequent transfection of cells is required or cannot be avoided. One example would be the repeated transfection of mRNAs coding transcription factors used for reprogramming of human fibroblasts and other differentiated cells to pluripotent stem cells [42, 43]. In another study, Michel et al. have investigated repeated co-transfection of EGFP and B18R mRNA in fibroblasts. They found that the presence of B18R significantly increased protein expression and in contrast reduced interferon expression over repeated transfection of cells [44]. Differential expression kinetics mediated by successive transfection could also be used to gain mechanistic biological insights. In this respect, Fan et al. elucidated the pharmacology of receptors and suggested that the interaction between the two parts of a receptor occurred constitutively if co-transfected, but not when expression was temporally separated, so they concluded that the mechanism of hetero-oligomers formation was likely co-translational [45].

Aside from some limitations, parallel co-delivery and successive delivery provide most flexibility for delivery of two nucleic acid entities with distinct properties. For instance, when dealing with complex gene networks, overexpression of one or even several genes needs to be concurrent with knock-down of other genes. Mimicking such conditions, by comparison of different co-transfection methods for co-delivery of siRNA along with IVT-mRNA, suggested that integrated co-transfection resulted in the highest rates of mCherry overexpression and EGFP knock-down, despite the low mCherry intensity at day 3. In parallel co-transfection, however, the knock-down efficiency was rather low, which proved that there is not necessarily a formulation for a carrier which is optimal for different types of cargos. In this particular case, when transfected with Lipo2000, siRNA was more efficiently delivered for EGFP knock-down. This finding is consistent with a study by Miller et al., in which they reported that co-delivery of single-guide RNA (sgRNA) with 100 nucleotides and Cas9 mRNA with 4500 nucleotides was most effective both for in vitro and in vivo CRISPR/Cas gene editing, when separate zwitterionic amino lipids with distinct features were administrated. Moreover, by evaluation of successive delivery of sgRNA and siRNA, they have suggested kinetically different maximum effect for two entities due to their various functional mechanisms, supporting the need for sequential delivery [14]. In another recent study, reprogramming of primary human fibroblasts to iPSCs was efficiently performed by successive transfection of miRNA and mRNA [17].

In summary, the quantitative measurements of the co-transfection rates on the single-cell level in our study revealed the extent to which outcomes depend on the delivery scheme and strategy followed. The results emphasize the notion that efficient co-delivery protocols have to be designed on a one-by-one case. Our findings can serve as a guideline for future researches for selecting the appropriate co-delivery method matched to key experimental requirements according to the specific biological questions to be addressed. Moreover, the quantitative evaluation of complex patterns of cell transfection resulting from the different approaches can support studies directed towards predictive modeling of the transfection process, including the identification of chemical and physical carrier criteria to be implemented for the most efficient cargo loading to ensure co-delivery of nucleic acids.

## Electronic supplementary material

ESM 1 (PDF 259 kb)

## Data Availability

The datasets generated during and/or analyzed during the current study are available from the corresponding author on a reasonable request.
