# Supplementary material for: Strategies for simultaneous and successive delivery of RNA
Source: J Mol Med (Berl). 2020 Nov 4;98(12):1767–79. doi: 10.1007/s00109-020-01956-1 (PMC7679312; doi:10.1007/s00109-020-01956-1)
Supplement: Supplementary file 1 — (PDF 259 kb) [file 109_2020_1956_MOESM1_ESM.pdf]

Strategies for Simultaneous and Successive Delivery of RNA

Hanieh Moradian[1, 2, 3], Andreas Lendlein[1, 2, 3], Manfred Gossen[1, 2, *]

1 Institute of Biomaterial Science, Helmholtz-Zentrum Geesthacht, 14513 Teltow, Germany
2 Berlin-Brandenburg Center for Regenerative Therapies (BCRT), 13353 Berlin, Germany
3 Institute of Biochemistry and Biology, University of Potsdam, Potsdam, Germany

* Corresponding Author, email address: manfred.gossen@hzg.de

A) Supplementary Materials and Methods

Evaluation of Encapsulation Efficiency

mRNA/carrier complexes were prepared as described in "Materials and Methods; Co-delivery of mRNA to HeLa cells via a lipid- or a polymeric-based carrier", using EGFP mRNA. The protocol for quantification of encapsulation efficiency is adapted from [1]. Different ratios of transfection reagent/mRNA (v/w) were tested, keeping mRNA concentration constant. Upon formation of complexes, samples were diluted in 1x TE assay buffer (10 mM Tris-HCl, 1 mM EDTA, pH 7.5) to reach an equal amount of mRNA for all samples in 100 µL of buffer. Subsequently, 100 µL of 200-fold diluted Quant-iT RiboGreen RNA reagent (ThermoFisher Scientific, Germany) was added to all samples. In parallel, equal volume of the same samples were dissociated by incubation with 5 µL of either 10% Triton X-100 for LipoMM complexes, or heparin solution (5mg·mL^{-1} in TE buffer) for ViroR complexes. Samples were incubated for 20 min at 37 °C in the dark. TE buffer only was used as blank. Fluorescence was measured using a Tecan Infinite 200 PRO plate reader with an excitation wavelength of 485 nm and emission wavelength of 535 nm. The percent of encapsulation efficiency was determined using the following formula:

$$\{ ([E(Diss.S) – E(B)] - [E(S) – E(B)]) / [E(Diss.S) – E(B)] \} \times 100$$

E(Diss.S) refers to emission of dissociated samples, i.e. total mRNA, E(S) describes emission of main samples, i.e. free mRNA in solution, and E(B) represents emission of blank at 535 nm. All samples were prepared as individually prepared triplicates.

Measurement of Physicochemical Properties of mRNA/carrier Complexes

The mRNA/carrier complexes, i.e. lipoplexes for LipoMM as well as polyplexes for ViroR, were evaluated in terms of size and zeta potential as two key physicochemical features. In order to elucidate the impact of mRNA type on final particles features, complexes were prepared with distinct types of mRNA, i.e. mRNA coding EGFP as well as mRNA coding mCherry. The size, polydispersity index (PDI) and zeta potential of complexes was determined by dynamic light scattering (DLS) via a ZetasizerNano from SZ instruments (Malvern Instruments, Herrenberg, Germany). Samples were diluted in 10 mM HEPES buffer with 1:4 and 1:10 dilution factor for LipoMM, and ViroR, respectively. The measurements were performed at 25 °C in disposable cuvettes.

B) Supplementary Data

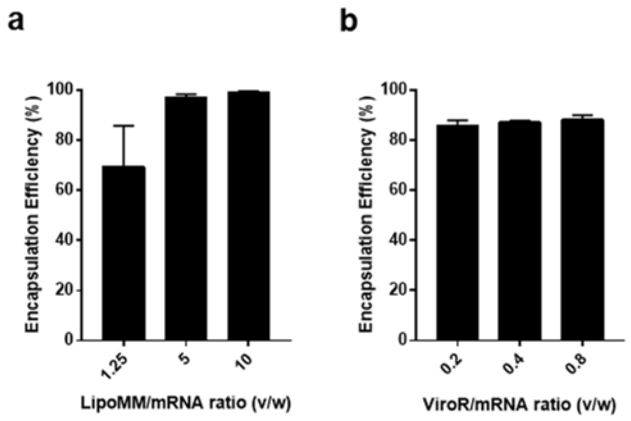

Supplementary Fig. 1 Encapsulation efficiency for carrier systems used for mRNA delivery **a** Lipofectamine messenger max (LipoMM) and **b** Viromer Red (ViroR). Three carrier/mRNA ratios were tested for both LipoMM and ViroR (µl·µg^{-1}). The middle ratio transfection reagent/mRNA is that used throughout the transfection experiments. Values are presented as mean ± SD, n=3. Error bars indicate SD.

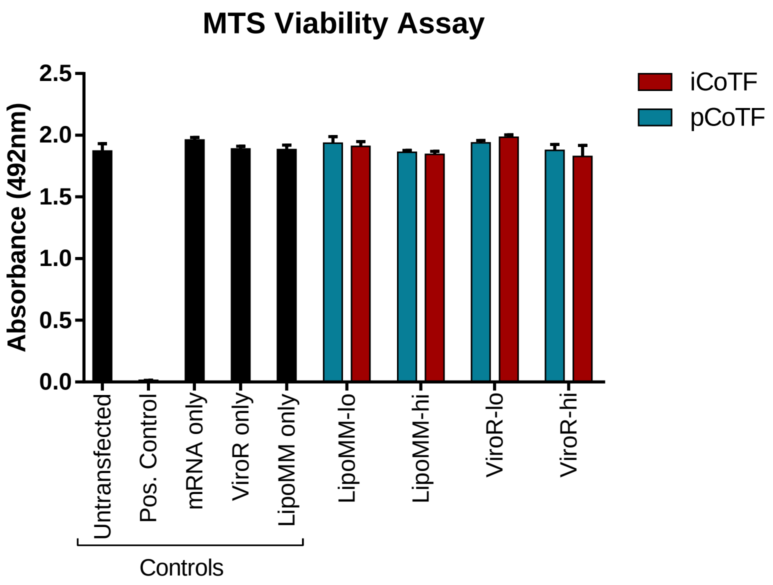

Supplementary Fig. 2 Viability assay measured with MTS assay, no significant differences were observed between the two co-delivery methods, i.e. iCoTF and pCoTF, neither for low nor for high mRNA doses ($p>0.05$). Values are presented as mean ± SD, n≥3. Error bars indicate SD. (iCoTF: integrated co-transfection, pCoTF: parallel co-transfection)

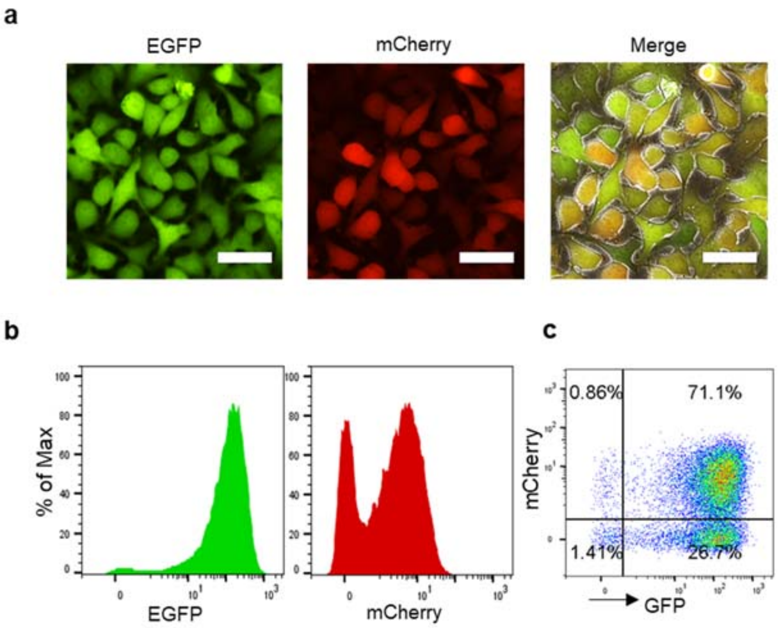

Supplementary Fig. 3 Successive transfection of IVT-mRNAs; cells were transfected with EGFP at first step and with mCherry mRNA at day 1 and were analyzed at day 2 **a-c**. Fluorescent images depicted as single channels and merged with phase contrast (bar =50 µm) **a**, flow cytometric analysis of cells; histograms of EGFP and mCherry **b** and density plot **c**.

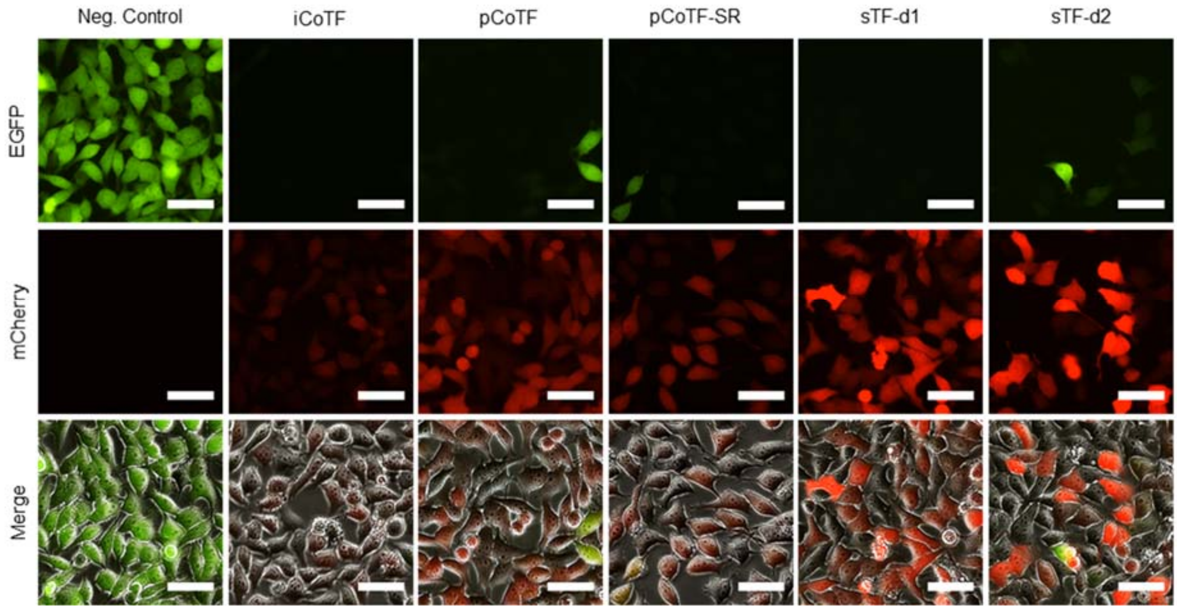

Supplementary Fig. 4 Co-delivery versus successive of siRNA and IVT-mRNA in d2EGFP Hela cells; various methods evaluated by fluorescent microscopy imaging (bar =50 µm).

Supplementary Table 1. Particle size and zeta potential of the two carrier systems complexed with either EGFP or mCherry mRNA. Multiple comparison tests have shown no significant statistical difference between size and zeta values for complexes prepared with different mRNA types for each carrier type, i.e. p-values were greater than 0.05 for all cases.

Sample Name	Particle size		Zeta potential
	d (nm) ± SD	PDI ± SD	(mV) ± SD
LipoMM/ EGFP	515,90 ± 64,06	0,70 ± 0,14	-0,24 ± 0,27
LipoMM/ mCherry	497,94 ± 28,41	0,53 ± 0,10	-0,02 ± 0,09
ViroR/ EGFP	517,79 ± 40,97	0,11 ± 0,07	-0,09 ± 0,16
ViroR/ mCherry	511,93 ± 58,75	0,16 ± 0,07	-0,08 ± 0,51

References

1. Li B, Dong Y (2017) Preparation and Optimization of Lipid-Like Nanoparticles for mRNA Delivery. Methods in Molecular Biology (Clifton, NJ) 1632:207-217. doi:10.1007/978-1-4939-7138-1_13
